# Arginine/Lysine Residues in the Cytoplasmic Tail Promote ER Export of Plant Glycosylation Enzymes

**DOI:** 10.1111/j.1600-0854.2008.00841.x

**Published:** 2008-11-03

**Authors:** Jennifer Schoberer, Ulrike Vavra, Johannes Stadlmann, Chris Hawes, Lukas Mach, Herta Steinkellner, Richard Strasser

**Affiliations:** 1 Department of Applied Plant Sciences and Plant Biotechnology, Institute of Applied Genetics and Cell Biology, University of Natural Resources and Applied Life Sciences, BOKU-ViennaMuthgasse 18, 1190 Vienna, Austria; 2 Department of Chemistry, University of Natural Resources and Applied Life Sciences, BOKU-ViennaMuthgasse 18, 1190 Vienna, Austria; 3 School of Life Sciences, Oxford Brookes UniversityHeadington, Oxford, OX3 0BP, UK

**Keywords:** COPII, cytoplasmic tail, ER exit, glycosylation, glycosyltransferase, Golgi targeting

## Abstract

Plant *N*-glycan processing enzymes are arranged along the early secretory pathway, forming an assembly line to facilitate the step-by-step modification of oligosaccharides on glycoproteins. Thus, these enzymes provide excellent tools to study signals and mechanisms, promoting their localization and retention in the endoplasmic reticulum (ER) and Golgi apparatus. Herein, we focused on a detailed investigation of amino acid sequence motifs present in their short cytoplasmic tails in respect to ER export. Using site-directed mutagenesis, we determined that single arginine/lysine residues within the cytoplasmic tail are sufficient to promote rapid Golgi targeting of Golgi-resident *N*-acetylglucosaminyltransferase I (GnTI) and α-mannosidase II (GMII). Furthermore, we reveal that an intact ER export motif is essential for proper *in vivo*function of GnTI. Coexpression studies with Sar1p provided evidence for COPII-dependent transport of GnTI to the Golgi. Our data provide evidence that efficient ER export of Golgi-resident plant *N*-glycan processing enzymes occurs through a selective mechanism based on recognition of single basic amino acids present in their cytoplasmic tails.

The plant Golgi apparatus consists of numerous separate stacks of cisternae, which are distributed throughout the cytoplasm and often associated with the endoplasmic reticulum (ER) (1). In many plant cell types, the Golgi stacks are highly motile and move along the tubular ER network in a microfilament-dependent way (2–4). In contrast to animal cells, exchange of cargo between these two organelles does not involve an ER-to-Golgi intermediate compartment and is assumed to occur either by specific transport vesicles or by permanent or transient tubular connections (5,6). The dynamic and close association of the plant ER and Golgi is different from that of mammalian and yeast cells and might be critical for mediating protein trafficking between the ER and Golgi (5,7,8).

The plant Golgi apparatus is not only a central organelle for protein sorting within the endomembrane system but plays also a major role in the biosynthesis of cell wall polysaccharides and maturation of glycoproteins (9). *N*-glycosylation is an abundant covalent protein modification in all eukaryotic cells. The core oligosaccharide, which is transferred to nascent proteins from a lipid-linked precursor, is extensively modified by removal and addition of sugar residues in the ER and subsequently in the Golgi apparatus (10,11). *N*-glycan processing is performed by a number of ER- and Golgi-resident glycosidases and glycosyltransferases, which are thought to act on cargo glycoproteins in a highly ordered fashion in a kind of assembly line. Thus, the subcellular localization of these enzymes together with their *in vivo*substrate specificity determines the carbohydrate structures of glycoproteins transported through the secretory pathway. How the ER and Golgi maintain the organization of these *N*-glycan processing enzymes is not well understood.

A number of plant *N*-glycan processing enzymes have been identified and characterized recently. Evidence for Golgi localization has been provided for some of them, reflecting their function in the processing pathway (3,12–16). Most of the characterized glycosidases and glycosyltransferases are typical type II membrane proteins, consisting of a short N-terminal cytoplasmic tail, a single transmembrane domain and a stem region (together the CTS region) orienting a catalytic domain into the Golgi lumen. It has been shown that important information for concentration of these type II membrane proteins in the Golgi is present in the CTS region, without any detectable contribution from the luminal catalytic domains (3,12–16). Although the importance of the CTS region for proper intracellular targeting of plant *N*-glycan processing enzymes is well documented, the role of the individual CTS domains for ER exit and Golgi concentration remains to be established.

In mammalian and yeast cells, protein transport between the ER and Golgi involves the vesicular coat protein complexes COPI and COPII. The COPII machinery is required for anterograde trafficking outside the ER by actively sorting secretory proteins into COPII transport vesicles, which is the assumed default route from the ER to the Golgi complex in mammals (17). Although the existence of COPII vesicles remains to be unequivocally shown in plants, homologues of COPII proteins have been identified, and COPII-dependent ER export has been demonstrated for soluble and transmembrane proteins (18–21).

Different classes of targeting signals have been identified in the cytoplasmic domains of transmembrane proteins in yeast and mammals. These motifs include cytoplasmically exposed diacidic, dihydrophobic and dibasic motifs (22,23). In contrast, the specific signals and underlying mechanisms that promote ER exit and retention of the corresponding enzymes in the plant Golgi apparatus are still poorly understood. So far, it has been shown that diacidic motifs present in the cytoplasmic regions of the Golgi nucleotide sugar transporter GONST1 and CASP, a member of the golgin family, contribute to ER export of these proteins (24). Furthermore, mutation of a basic motif present in the cytoplasmic tail of prolyl 4-hydroxylase was found to impair its transport to the Golgi in tobacco BY2-cells (25). These studies suggest that the cytoplasmic portion of transmembrane proteins harbours important information for ER exit in plants.

For mammalian glycosyltransferases involved in glycolipid synthesis, it was found that the conserved dibasic amino acid motif in the N-terminal cytoplasmic tail binds directly to the small guanosine triphosphatase (GTPase) Sar1p, indicating that the export of these glycosyltransferases occurs through the formation of COPII vesicles at ER export sites (ERES) (23). In addition, it was found that interactions between Golgi-resident glycosyltransferases and COPII components regulate COPII coat assembly (26). For plants, it has been shown that Golgi localization of green fluorescent protein (GFP)-tagged rat α2,6-sialyltransferase (ST–GFP) (2) occurs in a COPII-dependent way (20), and studies on coexpression of a Sar1p isoform with ST–GFP resulted in increased recruitment of Sar1p to ERES in *Nicotiana tabacum*leaf epidermal cells (21). However, the detailed mechanisms that dictate ER exit and concentrate plant *N*-glycan processing enzymes in the Golgi have remained elusive so far.

In this work, we investigated whether the ER export of different Golgi-resident *N*-glycan processing enzymes is influenced by amino acid sequence motifs present in the cytoplasmic tail. Using a series of deletion mutants, we determined the protein domains required for efficient Golgi retention. Furthermore, using site-directed mutagenesis, we identified amino acid residues required for efficient ER export. For one enzyme, Golgi-resident *N*-acetylglucosaminyltransferase I (GnTI), the functional significance of proper Golgi targeting was tested by complementation studies in GnTI-deficient *Arabidopsis thaliana*plants. Coexpression of GnTI with Sar1p forms indicated the presence of COPII-dependent transport to the Golgi. Our data show that despite the unique structural characteristics of the plant ER–Golgi interface, the ER-to-Golgi transport mechanisms of glycosyltransferases seem to be similar in mammals and plants.

## Results

### Lumenal sequences of GnTI are not required for Golgi localization

**Figure 1:**
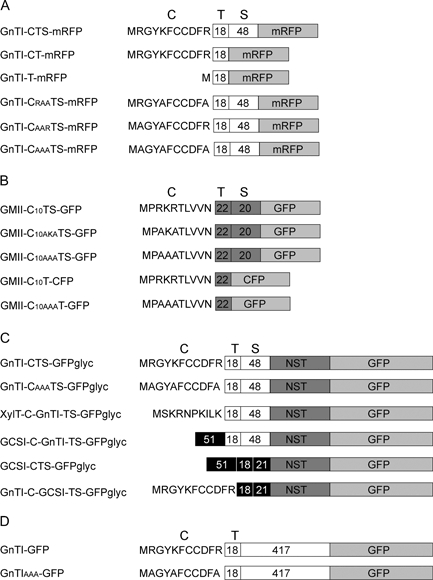
**A schematic representation of constructs used in this study.**All constructs were expressed under the control of the cauliflower mosaic virus 35*S*promoter. C, cytoplasmic tail; T, transmembrane domain; S, stem region; either the sequence or the number of amino acids is given for each domain; mRFP, monomeric red fluorescent protein. A) GnTI-CTS-mRFP, the 77 N-terminal amino acids (CTS region) of *N. tabacum*GnTI fused to mRFP; GnTI-CT-mRFP, the 29 N-terminal amino acids (CT region) of GnTI fused to mRFP; GnTI-T-mRFP, the transmembrane domain of GnTI fused to mRFP; GnTI-C_RAA_TS-mRFP, GnTI-C_AAR_TS-mRFP and GnTI-C_AAA_TS-mRFP, tail-mutated forms of GnTI-CTS-mRFP, where the basic amino acids present in the cytoplasmic tail were replaced by alanine residues. B) GMII-C_10_TS-GFP, the 52 N-terminal amino acids of *A. thaliana*Golgi α-mannosidase II consisting of a truncated cytoplasmic tail were fused to GFP; GMII-C_10AKA_TS-GFP and GMII-C_10AAA_TS-GFP, tail-mutated forms of GMII-C_10_TS-GFP, where the basic amino acids present in the cytoplasmic tail were replaced by alanine residues; GMII-C_10_T-GFP and GMII-C_10AAA_T-GFP, corresponding fusion proteins lacking the stem region. C) Wild type (GnTI-CTS-GFPglyc) or tail-mutated CTS region (GnTI-C_AAA_TS-GFPglyc) fused to the glycoprotein–GFP domain; NST: glycoprotein domain, 217 amino acids from the heavy chain of human IgG1 containing a single N-glycosylation site; XylT-C-GnTI-TS-GFPglyc, GnTI-C tail was replaced by *A. thaliana*β1,2-xylosyltransferase C-tail; GCSI-C-GnTI-TS-GFPglyc, GnTI-C tail was replaced by C-tail from *A. thaliana*GCSI; GCSI-CTS-GFPglyc, GnTI-CTS region was replaced by *A. thaliana*α-glucosidase I CTS region; GnTI-C-GCSI-TS-GFPglyc, the C-tail of GCSI was replaced by the C-tail of GnTI. D) GnTI-GFP, full-length *N. tabacum*GnTI fused to GFP; GnTI_AAA_-GFP, full-length tail-mutated GnTI form fused to GFP.

**Figure 2:**
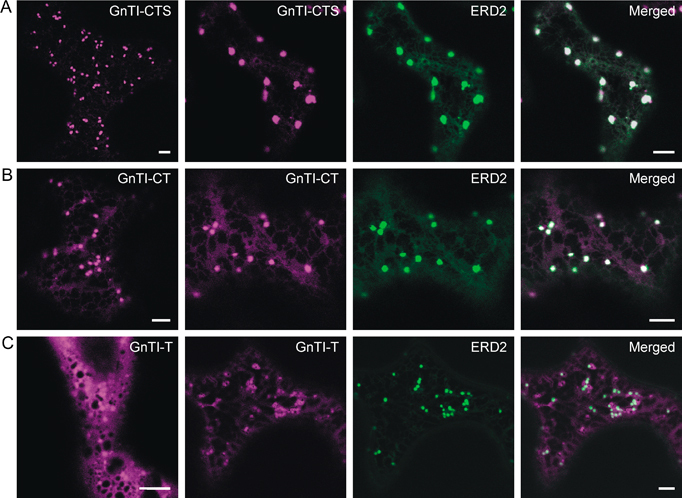
**The correct targeting of GnTI to its steady-state location relies on signals located in the cytoplasmic tail and transmembrane domain.**A) Confocal images of *N. benthamiana*leaf epidermal cells expressing GnTI-CTS-mRFP either alone or together with ERD2-GFP. The GnTI-CTS region targets mRFP predominantly to the Golgi. B) When expressed alone or coexpressed with ERD2-GFP, GnTI-CT-mRFP accumulates mainly in the Golgi, demonstrating that the luminal stem region is not needed for GnTI localization. C) In contrast, GnTI-T-mRFP highlights the cytoplasm and punctae of mostly unknown nature that only partially colocalize with ERD2-GFP. Scale bar = 5 μm for all images.

We have previously shown that the 77 N-terminal amino acids (CTS region) of *N. tabacum*GnTI consisting of a short cytoplasmic tail (11 amino acids), a single transmembrane domain (18 amino acids) and a lumenal stem region (48 amino acids) are sufficient for targeting of a reporter protein to the Golgi apparatus in plants (12). This finding was confirmed by others, who further truncated the putative stem region to seven residues and found that the remaining 36 N-terminal amino acids were still sufficient to target GFP to the Golgi apparatus (15). To test the relative contribution of the domains to *in vivo*localization in more detail, we generated constructs, where the GnTI CTS region was fused to the N-terminal part of monomeric red fluorescent protein (mRFP) and made deletions thereof (Figure 1A). These constructs were used for transient expression in *Nicotiana benthamiana*leaf epidermal cells. GnTI-CTS-mRFP was found predominantly in small motile bodies, resembling the Golgi apparatus (Figure 2A). The reticulate ER network was stained to a much lesser extent. This finding was confirmed by colocalization with the well-characterized ER/Golgi marker ERD2-GFP (2,4,27) (Figure 2A) and with the well-known Golgi marker ST–GFP (2,27) (data not shown) and is consistent with previous observations (12,15).

To analyse the effect of complete removal of the lumenal stem region on targeting of GnTI, the cytoplasmic tail and transmembrane domain were fused to mRFP (GnTI-CT-mRFP) and transiently expressed in *N. benthamiana*. GnTI-CT-mRFP was localized to punctate structures (Figure 2B), which colocalized with ERD2-GFP and resembled the structures observed with GnTI-CTS-mRFP. Finally, we removed the cytoplasmic tail of GnTI completely and analysed the distribution of the transmembrane domain-mRFP fusion (GnTI-T-mRFP). This fusion protein marked the cytoplasm and punctate structures of mostly unknown nature while only partially colocalizing with ERD2-GFP (Figure 2C). These data suggest that the cytoplasmic tail of GnTI is a major determinant of proper targeting to the Golgi. In this respect, GnTI is very similar to other plant *N*-glycosylation enzymes like *A. thaliana*Golgi α-mannosidase II (GMII) (14) and β1,2-xylosyltransferase (XylT) (13), where the transmembrane domain alone failed to provide Golgi localization of reporter constructs.

### A single arginine residue proximal to the transmembrane domain of GnTI is sufficient for Golgi localization

**Figure 3:**
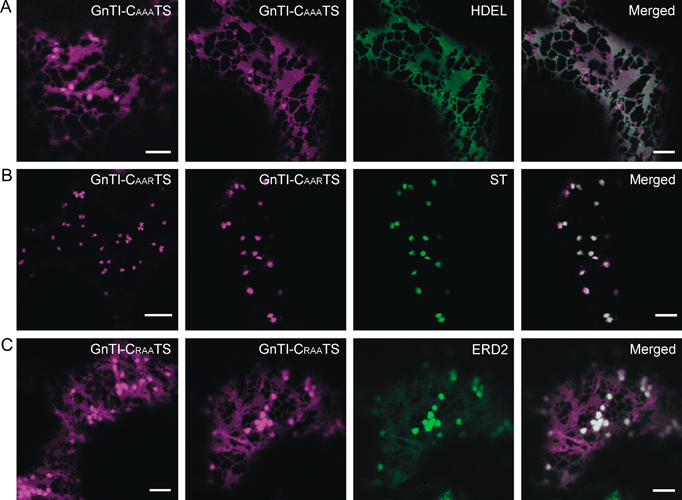
**Basic amino acids in the cytoplasmic tail of GnTI and their position relative to the transmembrane border are critical for efficient ER export.**All tail-mutated forms of GnTI were transiently expressed in *N. benthamiana*leaf epidermal cells either alone or in combination with ER and Golgi markers. A) Expression of GnTI-C_AAA_TS-mRFP alone or in combination with the ER marker GFP-HDEL. Upon mutation of all basic amino acids present in the cytoplasmic tail, most of the mutated protein remains in the ER with only a minor fraction reaching the Golgi. B) When GnTI-C_AAR_TS-mRFP was expressed in combination with ST–GFP, the former is confined to punctate structures colocalizing with the Golgi marker. C) An RKR→RAA substitution gives intermediate results, with part of GnTI-C_RAA_TS-mRFP in the ER and part in the Golgi as shown by colocalization with ERD2-GFP. Scale bar = 5 μm for all images.

To further investigate the contribution of the cytoplasmic tail to Golgi localization, we decided to mutate residues, which might be involved in ER-to-Golgi transport or Golgi retention of GnTI. The cytoplasmic tail of GnTI (C: M**R**GY**K**FCCDF**R**, basic amino acid residues are shown in bold) contains three basic amino acids, which could promote ER exit, as demonstrated for mammalian glycosylation enzymes (23). To test this hypothesis, these basic amino acids were replaced by alanine residues and the CTS-mRFP fusion proteins with different mutated cytoplasmic tails (C_RAA_TS, C_AAR_TS and C_AAA_TS; Figure 1A) were expressed transiently in *N. benthamiana*. The chimeric protein lacking all three basic amino acids in the cytoplasmic tail (C_AAA_TS) displayed a predominant ER steady-state location with little Golgi labelling (Figure 3A), demonstrating their importance for efficient ER exit. In contrast, the construct harbouring only the arginine proximal to the transmembrane domain (C_AAR_TS) was targeted to the Golgi apparatus quite similar to the wild-type form (GnTI-CTS-mRFP) (Figure 3B), thus revealing that the presence of this residue is sufficient for proper Golgi targeting. Interestingly, the more distal arginine was less effective in this respect. The corresponding fusion protein (C_RAA_TS) was detected both in the ER and in the Golgi apparatus (Figure 3C), with a more pronounced labelling of the ER when compared with GnTI-CTS-mRFP (Figure 2A). Colocalization of the tail-mutated proteins with ST–GFP, GFP-HDEL or ERD2-GFP confirmed their ER and Golgi location, respectively.

To rule out that the ER accumulation of the tail-mutated form (C_AAA_TS) was caused by overexpression, we compared the expression levels in extracts 1–3 days after infiltration by immunoblotting using anti-red fluorescent protein (RFP) antibodies. A band of the expected size was clearly visible after 2 and 3 days for GnTI-CTS-mRFP and GnTI-C_AAA_TS-mRFP (Figure S1), but a much longer exposure of the blot (data not shown) was required to detect this band after day 1. However, confocal microscopy data showed the typical localization pattern for these proteins already after 1 day, demonstrating that ER accumulation of GnTI-C_AAA_TS-mRFP is not a consequence of overexpression.

### Basic amino acids in the cytoplasmic tail of other glycosylation enzymes are important for proper Golgi localization

**Figure 4:**
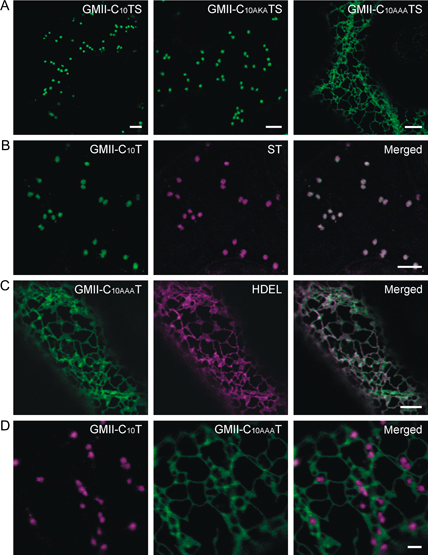
**Golgi concentration of GMII depends on basic residues within the N-terminal targeting-determining region.**A) Confocal image of a *N. benthamiana*cell expressing GMII-C_10_TS-GFP. The basic amino acid motif (RKR) present in the truncated cytoplasmic tail of GMII is able to direct the fusion protein outside the ER and concentrate it in the Golgi apparatus. Replacement of both arginines with alanines does not affect export of GMII-C_10AKA_TS-GFP from the ER. Mutation of the entire motif induced accumulation of GMII-C_10AAA_TS-GFP in the ER. Scale bar = 5 μm. B) Coexpression of GMII-C_10_T with the Golgi marker ST–mRFP demonstrates that the punctuate structures labeled by GMII are Golgi bodies, as shown in the merged image. Scale bar = 5 μm. C) In contrast, the corresponding tail-mutated GMII-C_10AAA_T protein fails to exit from the ER. Note the colocalization with the ER marker mRFP-HDEL in the merged image. Scale bar = 5 μm. D) Coexpression of wild-type GMII-C_10_T and tail-mutated GMII-C_10AAA_T demonstrates that the steady-state location of the chimeric wild-type GMII protein is the Golgi apparatus and the mutated fusion protein remains in the ER. Scale bar = 2 μm.

We have previously shown that a truncated cytoplasmic tail (C_10_) of GMII containing only 10 amino acids fused to its transmembrane domain is sufficient for Golgi targeting and retention (14). To analyse whether the basic amino acids present in the C_10_ tail of GMII play a similar role as observed for the positively charged amino acids of GnTI, chimeric proteins consisting of the C_10_ tail (C_10_: MP**RKR**TLVVN; basic amino acid residues are shown in bold; Figure 1B), transmembrane and stem regions were fused to GFP (GMII-C_10_TS-GFP), the basic amino acids were replaced by alanine residues and the proteins transiently expressed in *N. benthamiana*leaf epidermal cells. GMII-C_10AKA_TS-GFP with one remaining basic amino acid displayed a Golgi distribution like GMII-C_10_TS-GFP (Figure 4A). Further exchange of the lysine residue (GMII-C_10AAA_TS-GFP) resulted in a predominant ER steady-state localization (Figure 4A), as observed for GnTI-C_AAA_TS-mRFP. Removal of the stem region did not have any influence on the ER distribution of the tail-mutated GMII fusion protein (GMII-C_10AAA_T-GFP). Near perfect colocalization of GMII-C_10_T-cyan fluorescent protein (CFP) with ST–mRFP (Figure 4B) as well as of GMII-C_10AAA_T-GFP with the ER marker mRFP-HDEL (Figure 4C) and clear differences in the localization of GMII-C_10_T-CFP with GMII-C_10AAA_T-GFP (Figure 4D) confirmed the result. Our data demonstrate that similar to GnTI, at least one basic amino acid in the cytoplasmic tail plays a key role in ER exit or Golgi retention of GMII. In line with this observation, exchange of the four basic amino acids in the cytoplasmic tail of another Golgi-located glycosyltransferase, XylT (C: MS**KR**NP**K**IL**K**, basic amino acid residues are shown in bold), with alanine residues led to accumulation of a GFP fusion protein in the ER (Figure S2). It has been recently shown that the presence of the proximal lysine is sufficient for Golgi targeting of a XylT fusion protein (28). Taken together, these results demonstrate the importance of individual basic amino acids in the cytoplasmic tails of plant glycosylation enzymes for ER exit or active Golgi targeting.

### Removal of basic amino acids in the cytoplasmic tail of GnTI does not alter the membrane topology of the protein

**Figure 5:**
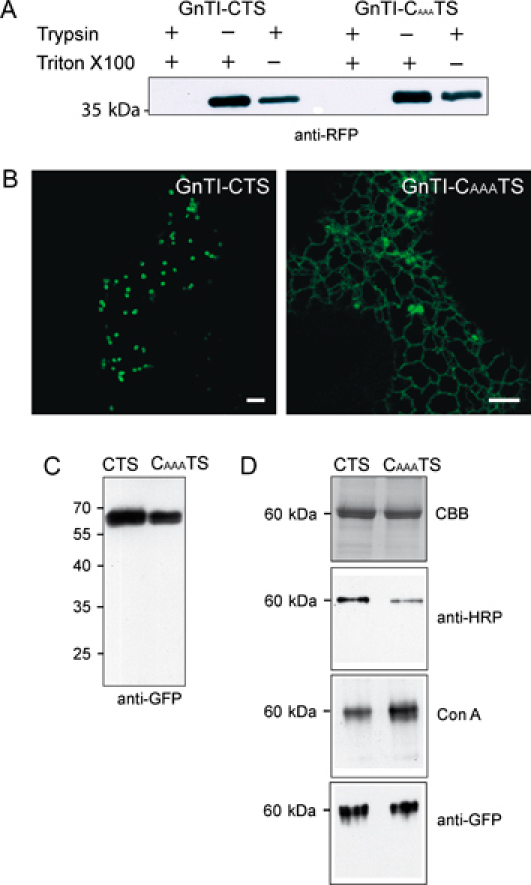
**The topology is not altered in the tail-mutated GnTI fusion protein.**A) Microsomal fractions were isolated from infiltrated leaves, treated with trypsin (0.5 μg) in the absence or presence of 1% Triton-X-100 and analysed by western blot using anti-RFP antibody. B) Confocal images of cells expressing either GnTI-CTS-GFPglyc or GnTI-C_AAA_TS-GFPglyc in *N. benthamiana*leaf epidermal cells. GnTI-CTS-GFPglyc concentrates predominantly in the Golgi apparatus, whereas most of the mutated form GnTI-C_AAA_TS-GFPglyc remains in the ER. Scale bar = 5 μm. C) Immunoblot analysis of GnTI-C_AAA_TS-GFPglyc and GnTI-CTS-GFPglyc expression: proteins were extracted from *N. benthamiana*leaves, and detection was performed with anti-GFP antibodies. D) SDS–PAGE and protein blot analysis of purified GnTI-C_AAA_TS-GFPglyc and GnTI-CTS-GFPglyc proteins; CBB, Coomassie-stained SDS–PAGE gel; anti-HRP, immunoblot, detection with anti-HRP antibody; Con A, lectin blot with concanavalin A; anti-GFP, immunoblot, detection with anti-GFP antibody.

It has been shown that the orientation of eukaryotic membrane proteins correlates with the charge difference of the flanking region, with the more positive portion of the protein facing the cytosol (29). To rule out that the observed difference in targeting of GnTI-C_AAA_TS is because of a change in the membrane orientation of the fusion protein caused by removal of three charged residues, we analysed the topology of the wild-type and ER-retained mRFP fusion proteins by protease protection assays. Isolated microsomal fractions from infiltrated leaves were treated with trypsin in the presence or absence of Triton-X-100 and analysed by SDS–PAGE and western blot using anti-RFP antibodies (Figure 5A). Trypsin treatment in the presence of detergents led to the quantitative degradation of both chimeric proteins. In the absence of Triton-X-100, the two mRFP fusion proteins were equally resistant to trypsin digestion, indicating that the exchange of the three basic amino acids in the cytoplasmic tail of GnTI with alanine did not alter the membrane orientation.

To provide further evidence for correct topology of the tail-mutated GnTI protein, we generated a glycoprotein reporter by exchanging mRFP with GFP and inserting a protein fragment with a single *N*-glycosylation site derived from the human immunoglobulin G1 (IgG1) heavy chain between the GnTI-CTS region and the fluorescent protein (Figure 1C). The resulting proteins GnTI-CTS-GFPglyc and the tail-mutated form GnTI-C_AAA_TS-GFPglyc displayed predominantly Golgi and ER steady-state localization, respectively, when transiently expressed in *N. benthamiana*leaf epidermal cells (Figure 5B). Coexpression with their mRFP counterparts showed perfect colocalization of the wild-type proteins (GnTI-CTS-mRFP/GnTI-CTS-GFPglyc) and mutated forms (GnTI-C_AAA_TS-mRFP/GnTI-C_AAA_TS-GFPglyc), respectively, and different localization when a wild-type form was coexpressed with a mutated one (GnTI-CTS-mRFP/GnTI-C_AAA_TS-GFPglyc or GnTI-C_AAA_TS-mRFP/GnTI-CTS-GFPglyc) regardless of the used fluorescent protein tag (data not shown).

Correct topology of the chimeric proteins, which differ only in their cytoplasmic tail, will orient the chimeric protein with the *N*-glycosylation site facing the ER lumen. Thus, transfer of the oligosaccharide precursor can occur, while the opposite orientation with the chimeric glycoprotein reporter in the cytosol would not result in any *N*-glycosylated protein. Western blot detection of proteins extracted from infiltrated leaves showed a band of expected size (60.4 kDa without accounting for the *N*-glycan moiety; Figure 5C) for both GnTI-CTS-GFPglyc and GnTI-C_AAA_TS-GFPglyc without any detectable amounts of free GFP or other degradation products. After affinity purification, a single discrete band was detectable on Coomassie-stained SDS–PAGE gels and immunoblots using anti-GFP antibodies (Figure 5D). Importantly, both purified proteins reacted with antibodies recognizing complex plant *N*-glycans (anti-HRP) and the mannose-binding lectin concanavalin A (Con A), indicating that both proteins are *N*-glycosylated and thus have proper orientation. Hence, we exclude that the observed difference in localization of the tail-mutated chimeric GnTI protein is triggered by a failure to acquire a type II membrane topology.

### The tail-mutated GnTI fusion protein shows a quantitative block in ER exit

**Figure 6:**
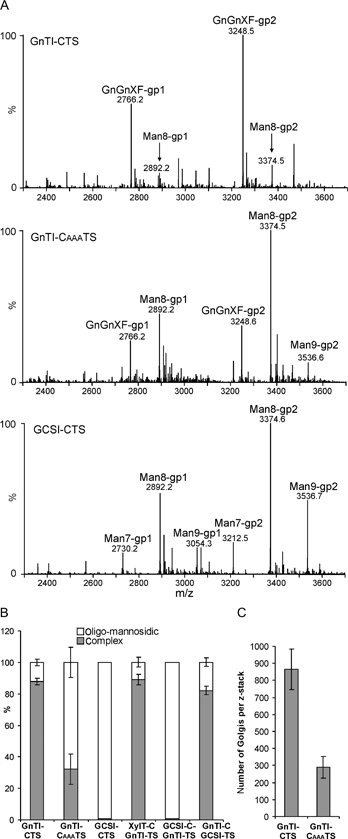
**LC-ESI-MS of glycoreporter fusion proteins.**A) Mass spectra of glycopeptides (−1, EEQYNSTYR; −2, TKPREEQYNSTYR) derived from the glycoprotein part of GnTI-CTS-GFPglyc, GnTI-C_AAA_TS-GFPglyc and GCSI-CTS-GFPglyc are shown. Man8, oligo-mannosidic *N*-glycan, indicative of ER retention; GnGnXF, complex *N*-glycan, processed in the Golgi apparatus (see http://www.proglycan.com for an explanation of *N*-glycan abbreviations). For all samples, 500 ng of purified protein were analysed. B) Quantification of the oligo-mannosidic and complex *N*-glycan peaks present in the mass spectra shown in (A) and of purified proteins GCSI-C-GnTI-TS-GFPglyc, XylT-C-GnTI-TS-GFPglyc and GnTI-C-GCSI-TS-GFPglyc. The values represent means ± SD of three independent experiments. C) Quantification of Golgi stacks present in GnTI-CTS-GFPglyc- and GnTI-C_AAA_TS-GFPglyc-expressing leaves. Values are mean ± SD of three independent experiments.

The observed differences in localization of the tail-mutated form of GnTI could either be the result of an impaired ER exit or because of an increased retrograde transport from the Golgi back to the ER. To test these two possibilities, we made use of the glycoprotein reporter (Figure 1C), where the CTS region can be exchanged and then effects on localization monitored by changes in the *N*-glycan profile. The presence of oligo-mannosidic structures (like Man8) attached to the glycoprotein is a hallmark of retention in the ER, while forward movement to the Golgi will lead to processing of *N*-glycans and the formation of complex *N*-glycans carrying β1,2-xylose and core α1,3-fucose residues (main glycoform: GnGnXF). The analysis of the purified chimeric GnTI-CTS-GFPglyc proteins by western blot with anti-HRP antibodies and Con A already indicated differences in their content of complex and oligo-mannosidic *N*-glycans. The signal with anti-HRP antibody was stronger for the wild-type form, while Con A gave the opposite result when equal amounts of protein were analysed (Figure 5D). To analyse the *N*-glycosylation pattern in detail, liquid chromatography-electrospray ionization-mass spectrometry (LC-ESI-MS) was performed on tryptic peptides of both purified proteins. In the case of GnTI-CTS-GFPglyc, the major peak was found to be the complex *N*-glycan structure GnGnXF (Figure 6A). In contrast, GnTI-C_AAA_TS-GFPglyc was found to contain a large quantity of the oligo-mannosidic *N*-glycan Man8 and only minor amounts of GnGnXF. A quantification of the relative amounts of complex and oligo-mannosidic structures revealed significant differences between the wild type (GnTI-CTS-GFPglyc) and the mutated form (GnTI-C_AAA_TS-GFPglyc). The complex-type content of the wild-type form amounted to 88%, whereas the mutant form contained much less (32%) (Figure 6B). The distribution of the glycoforms is consistent with the reduced number (33%) of GFP-labelled Golgi stacks in cells expressing the tail-mutated form (Figure 6C). These data reflect the difference in subcellular targeting between the two proteins and demonstrate that most of the tail-mutated protein is retained in the ER and never reaches the Golgi. Thus, our finding suggests that the complete removal of basic amino acids in the cytoplasmic tail impairs the ER exit of GnTI.

As a control for complete retention of a glycoreporter protein in the ER, we expressed a construct where the N-terminal targeting region of *A. thaliana*α-glucosidase I (GCSI) (15) was fused to GFP (Figure 1C). The *N*-glycans of this ER-retained glycoreporter (GCSI-CTS-GFPglyc) were exclusively of the oligo-mannosidic type (Man7–9; Figure 6A), showing that this reporter is restricted to the ER.

### The cytoplasmic tails of glycosylation enzymes are sufficient to redirect ER- and Golgi-located proteins

**Figure 7:**
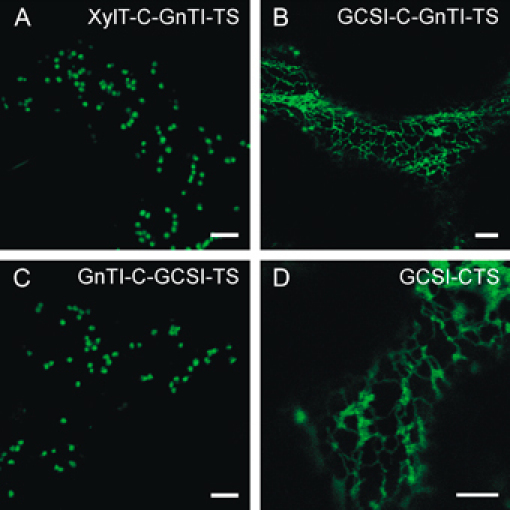
**Swapping the cytoplasmic tails of the three plant glycosylation enzymes GnTI, XylT and GCSI indicates that general subcellular targeting information is contained in their cytoplasmic tails.**A) Confocal image of a *N. benthamiana*leaf epidermal cell expressing XylT-C-GnTI-TS-GFPglyc. The exchange of the cytoplasmic tail of GnTI with the corresponding region of XylT does not alter the Golgi localization of GnTI. B) When the cytoplasmic tail of GnTI was swapped with the tail of GCSI, the chimeric protein GCSI-C-GnTI-TS-GFPglyc accumulated in the ER. C) Conversely, a chimeric protein bearing the GnTI cytoplasmic tail fused to the transmembrane stem region of GCSI is distributed to the Golgi apparatus. D) Combination with the CTS region of GCSI leads to accumulation of the fusion protein in the ER. Scale bar = 5 μm for all images.

Because we found that the cytoplasmic tail plays a critical role for localization of GnTI, GMII and XylT, we asked whether the complete exchange of the tail would affect their steady-state localization and redirect proteins from the Golgi to the ER. First, we tested a chimeric protein where the cytoplasmic tail of GnTI was replaced by the tail of XylT (Figure 1C). This chimeric protein (XylT-C-GnTI-TS-GFPglyc) behaved similarly to wild-type GnTI and was found predominantly in the Golgi apparatus (Figure 7A), carrying mainly complex *N*-glycans (Figure 6B). However, replacement of the cytoplasmic tail of GnTI with the cytoplasmic tail of GCSI (GCSI-C-GnTI-TS-GFPglyc; Figure 1C) resulted in relocation of the reporter protein to the ER (Figure 7B). The observed reticulate labelling of GCSI-C-GnTI-TS-GFPglyc was similar to that of GCSI-CTS-GFPglyc (Figure 7D) and comparable to previous observations (15). Like GCSI-CTS-GFPglyc, the GCSI-C-GnTI-TS-GFPglyc fusion protein contained more than 99% of oligo-mannosidic *N*-glycans (Figure 6B).

To test whether an ER-resident protein can be transported further down the secretory pathway to the Golgi apparatus, the cytoplasmic tail of GnTI was fused to the TS region of GCSI. The chimeric GnTI-C-GCSI-TS-GFPglyc protein was detected in the Golgi apparatus (Figure 7C), and consistent with the observed subcellular localization, the majority of its *N*-glycans was of the complex type (Figure 6B).

### GnTI with a mutated cytoplasmic tail is only partially functional *in vivo*

**Figure 8:**
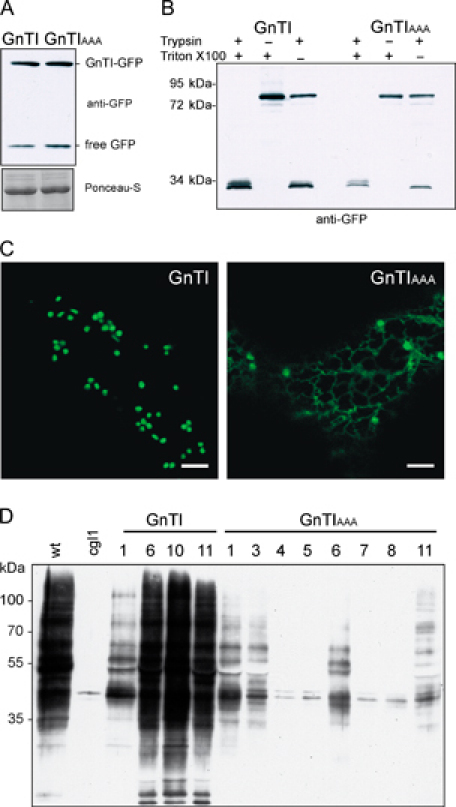
**Complementation of *A. thaliana cgl1*mutant with full-length GnTI proteins.**A) Comparison of wild-type and tail-mutated GnTI-GFP expression in *N. benthamiana*. Total proteins were extracted from leaves and subjected to SDS–PAGE under non-reducing conditions. Western blot analysis was performed using anti-GFP antibody. B) Protease protection assay to monitor the membrane topology of full-length GnTI-GFP proteins. Microsomal preparations were trypsin digested in the presence or absence of Triton-X-100. Western blot analysis was performed using anti-GFP. Note that the GFP degradation product in lanes 3 and 6 resulted from partially disrupted microsomal preparations. C) Confocal images of *N. benthamiana*leaf epidermal cells expressing wild-type and tail-mutated full-length GnTI constructs. GnTI-GFP highlights mainly the Golgi apparatus, whereas GnTI_AAA_-GFP predominantly accumulates in the ER; only a minor fraction still reaches the Golgi. Scale bar = 5 μm. D) Restoration of complex *N*-glycan formation in transgenic *A. thaliana cgl1*mutants. Extracted proteins (15 μg) were separated by SDS–PAGE and detected by immunoblotting using antibodies directed against β1,2-xylose- and core α1,3-fucose-containing complex *N*-glycans (anti-HRP); wt, untransformed Col-0 wild-type plant; *cgl1*, untransformed *cgl1*mutant; 1–11, *cgl1*mutants transformed either with GnTI-GFP (GnTI) or with GnTI_AAA_-GFP (GnTI_AAA_).

To analyse whether mutations in the cytoplasmic tail of GnTI lead to differences in the *in vivo*functionality because of altered localization of the enzyme, full-length wild type and mutated GnTI (C_AAA_) with GFP fused to the C-terminus (Figure 1D) were expressed in *A. thaliana cgl1*plants, which lack an active GnTI enzyme and produce only oligo-mannosidic *N*-glycans (30,31). We reasoned that a functional GnTI protein, which is targeted to the plant Golgi, would restore the formation of complex *N*-glycans in the transformed mutants, while the mutated form would display less complementation because of the ER retention. Transient expression in *N. benthamiana*leaf epidermal cells confirmed the expression of both constructs without any obvious differences in the levels of the full-length proteins and their endogenous degradation products (free GFP; Figure 8A). Protease protection assays were performed to obtain information about the membrane orientation of the two full-length GnTI forms. Incubation of microsomal preparations from GnTI-GFP or GnTI_AAA_-GFP expressing *N. benthamiana*leaves with trypsin demonstrated a similar resistance of the two fusion proteins to proteinase treatment, whereas the presence of Triton-X-100 resulted in either case in complete degradation (Figure 8B). Thus, GnTI_AAA_-GFP displays a type II membrane orientation like the wild-type form. Moreover, the subcellular location of the full-length fusion proteins was identical to their GnTI-CTS forms (Figure 8C and Figure S3), which is consistent with the previous findings that sequence motifs present in the lumenal catalytic domain do not contribute to Golgi localization of plant GnTI. Subsequently, stable *A. thaliana*lines were generated by floral dipping of *cgl1*plants, and for each construct, 20 lines were propagated. As expected, analysis of *cgl1*plants expressing full-length wild-type GnTI-GFP clearly showed significant complementation of the mutation, as evident by the presence of a strong staining signal with the anti-HRP antibody in all tested lines (Figure 8D). However, analysis of transgenic *cgl1*plants expressing the full-length tail-mutated form of GnTI (C_AAA_) resulted in a weak staining signal in most of the lines, and some lines did not show any staining. These findings were confirmed by matrix-assisted laser desorption ionization-time of flight-mass spectrometry (MALDI-TOF-MS) analysis of total *N*-glycans isolated from transformed *cgl1*plants, which showed the presence of complex-type *N*-glycans for wild-type GnTI, whereas the tail-mutated form produced only oligo-mannosidic structures (Figure S4). Taking these data together, we conclude that a GnTI protein, which lacks basic amino acids in its cytoplasmic tail, is at best partially functional *in vivo*.

### Expression of GnTI can trigger Sar1p accumulation at ERES

**Figure 9:**
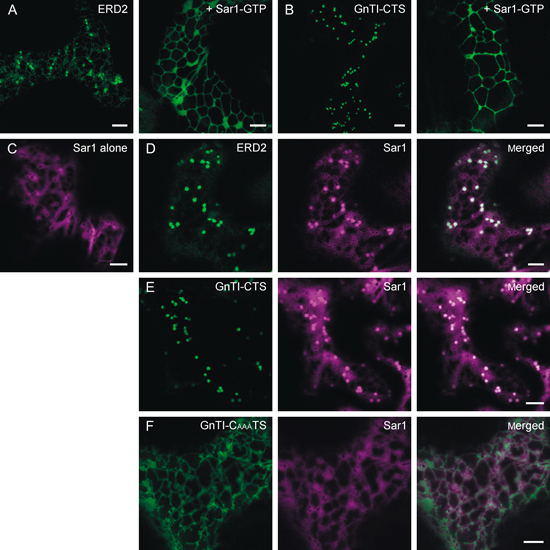
**Overexpression of GnTI leads to recruitment of the GTPase Sar1p onto ER membranes.**A and B) Coexpression of the dominant-negative mutant Sar1[H74L]p with either the Golgi marker ERD2 (A) or GnTI (B) in *N. benthamiana*leaf epidermal cells. A) Upon coexpression of ERD2-GFP with the untagged GTP-locked Sar1p mutant, fluorescence of ERD2-GFP did not accumulate in bright Golgi stacks anymore but was located in the ER. B) Similarly, when GnTI-CTS-GFP was expressed together with the mutant Sar1p construct, a pronounced ER staining was induced. C) Wild-type Sar1-YFP displays a cytoplasmic distribution when expressed in *N. benthamiana*leaf epidermal cells. D) Upon coexpression of Sar1-YFP and the Golgi marker ERD2-GFP, Sar1-YFP maintained a cytoplasmic staining and in addition, concentrated at defined punctate structures characteristic of ERES. Note the colocalization of Golgi bodies with ERES highlighted with Sar1-YFP. E) When GnTI-CTS-GFP was overexpressed in the presence of Sar1-YFP, Sar1-YFP showed cytoplasmic staining and bright spots colocalizing with Golgi bodies labeled with GnTI-CTS-GFP. F) Coexpression of GnTI-C_AAA_TS-GFP with Sar1-YFP did not result in any accumulation of Sar1-YFP in punctate structures. YFP fluorescence was only detected in the cytoplasm, whereas the tail-mutated GnTI construct showed its characteristic ER staining. Scale bar = 5 μm for all images.

In mammalian cells, the ER export of glycosyltransferases depends on the interaction of basic amino acids in the cytoplasmic tail with the small GTPase Sar1p (23), which is responsible for initiation of COPII coat assembly at ERES (22). Having established that the tail-mutated form of GnTI is significantly retained in the ER, we next wanted to determine whether the ER export of GnTI was COPII dependent. We expressed the untagged GTP-locked mutant version of Sar1p (Sar1[H74L]p) (20) together with GnTI-CTS-GFP in *N. benthamiana*leaf epidermal cells. As a control, we investigated the effect of the GTP-locked Sar1p mutant on the subcellular localization of ERD2-GFP (Figure 9A). The expression of the GTP-locked Sar1p form resulted in concentration of GnTI-CTS-GFP in the ER (Figure 9B), being consistent with previous observations demonstrating that Sar1[H74L]p causes the accumulation of membrane-bound Golgi marker proteins like ERD2-GFP (Figure 9A) or ST–GFP in the ER (18–20).

To test if expression of the wild-type GnTI fluorescent fusion protein could recruit Sar1p to ERES, as was shown for ERD2-GFP and ST–GFP (21), we coexpressed GnTI-CTS-GFP with wild-type Sar1-yellow fluorescent protein (YFP). Cytoplasmic labelling was visible in cells expressing Sar1-YFP alone **(**Figure 9C), whereas coexpression of Sar1-YFP and ERD2-GFP resulted in the appearance of bright punctate structures highlighted with Sar1-YFP (Figure 9D) as observed previously (5,21). Coexpression of GnTI-CTS-GFP together with Sar1-YFP resulted in the formation of similar punctate structures (Figure 9E), as observed for ERD2-GFP. In contrast, expression of the tail-mutated GnTI form did not result in any appearance of punctate structures (Figure 9F). These data indicate that the ER-to-Golgi transport of GnTI can be blocked by coexpression with Sar1[H74L]p and suggest the involvement of basic amino acids present in the cytoplasmic tail of GnTI in Sar1p recruitment to ERES.

## Discussion

The molecular mechanisms and signals for protein trafficking between the ER and Golgi apparatus are already fairly well characterized in mammals (22,23,32), whereas these processes are less well understood in plants (6,24,33). In this study, we aimed to answer the question to what extent the cytoplasmic tail of plant *N*-glycan processing enzymes contributes to ER exit and Golgi localization. Most of these *N*-glycan processing enzymes are predicted to display a type II membrane protein topology, with a short cytoplasmic tail, a single transmembrane domain and a stem or spacer region, which orients the catalytic domain into the ER or Golgi lumen (34). Their conserved topology and ordered arrangement in an assembly line along the early secretory pathway makes these enzymes valuable tools to investigate Golgi targeting and retention mechanisms. For mammalian glycosyltransferases, localization relies on contributions from several domains including signals in the lumenal domains (34–36). In plants, evidence for spatial arrangement of *N*-glycan processing enzymes has been provided, but the signals and underlying mechanisms are not well understood (15).

Our data from live-cell imaging demonstrate that the 29 N-terminal amino acids of *N. tabacum*GnTI, comprising the cytoplasmic tail and transmembrane domain, are sufficient for Golgi targeting and retention of a reporter protein. Being consistent with our data for *N. tabacum*GnTI, the N-terminal 31 amino acids of rabbit GnTI corresponding to the same domains were sufficient to concentrate a reporter protein in the Golgi apparatus in mammalian cells (37). Additional deletion of the cytoplasmic tail from *N. tabacum*GnTI seems to affect ER membrane insertion because we found a large portion of GnTI-T-mRFP located in the cytoplasm. Although the possible occurrence of free mRFP cannot be completely excluded, this finding is in contrast to mammalian glycosyltransferases, where the deletion of the cytoplasmic tail from different glycosyltransferases did not disturb the ER membrane insertion but impaired Golgi concentration (23). For these mammalian type II membrane proteins, it was shown that a conserved dibasic motif [RK]X[RK] present in the cytoplasmic tail proximal to the transmembrane domain is required for ER export. This motif is conserved in a large number of mammalian glycosyltransferases, including human GnTI (38). *N. tabacum*GnTI contains three basic amino acids in the 11 residues of its cytoplasmic tail. We provide evidence that a single arginine residue proximal to the transmembrane border, which is strictly conserved in plant GnTI proteins (39,40), is sufficient for concentration of GnTI in the Golgi like it has been proposed for mammalian glycosylation enzymes (23).

Our finding that basic amino acid residues in the cytoplasmic tail are critical for ER exit of GnTI and GMII is consistent with the observation that mutations of basic amino acids in the cytoplasmic tail of the type II membrane protein prolyl 4-hydroxylase from tobacco BY2 cells impaired its Golgi localization without affecting the transmembrane insertion and topology (25). However, the mutant form of prolyl 4-hydroxylase, which still contained one basic amino acid in the cytoplasmic tail proximal to the putative transmembrane domain, located to the ER. This indicates that not all type II membrane proteins have the same requirements for efficient ER export. Recently, Maruyama et al. (28) expressed a fluorescent protein fusion consisting of a mutated cytoplasmic tail and the transmembrane domain of XylT in *A. thaliana*seeds. Three basic amino acids were replaced with non-charged threonine residues, while the lysine residue proximal to the transmembrane domain was not altered. This fusion protein was detected mainly in the Golgi, which is in line with our observation for GnTI-C_AAR_TS. In contrast, our XylT-C_AAAA_TS protein with four alanines instead of the basic amino acids showed a reticular distribution pattern resembling ER. Other Golgi-resident plant *N*-glycan processing enzymes like soybean Golgi α-mannosidase I (3) and *A. thaliana*N-acetylglucosyminyltransferase II (41), core α1,3-fucosyltransferases (42), α1,4-fucosyltransferase (43) and β1,3-galactosyltransferase (16) contain at least one basic amino acid in their cytoplasmic tail.

When transiently expressed in *N. benthamiana*leaf epidermis, a significant fraction of GnTI-C_AAA_TS was still transported to the Golgi as evident from the number of GFP-positive Golgi stacks and the presence of complex *N*-glycans on the respective glycoreporter. The residual ER-to-Golgi transport of the tail-mutated GnTI protein could be mediated by signals other than basic amino acids, as it has been proposed for GONST1 and CASP (24), or through bulk flow. However, the tail-mutated full-length GnTI form almost completely failed to restore the N-glycosylation defect in the *A. thaliana cgl1*mutant. Thus, the removal of basic amino acids from the cytoplasmic tail of GnTI leads to a highly efficient ER export block which impairs the *in vivo*activity of the tail-mutated protein.

Accumulation of Golgi-resident glycosylation enzymes with altered cytoplasmic tails in the ER could be caused by exclusion from anterograde transport or increased recruitment from the Golgi by retrograde transport. Our finding of increased amounts of unprocessed oligo-mannosidic *N*-glycans in the tail-mutated ER-located GnTI protein (GnTI-C_AAA_TS-GFP) is clearly in favour of the first hypothesis. In line with this, the observed failure for ER export could be the result of a loss of interaction between the cytoplasmic tail of the cargo protein and the COPII components. For mammalian glycosyltransferases, direct *in vitro*interaction with the small GTPase Sar1p, which initiates COPII coat assembly, has been demonstrated (23). This interaction was lost by removal of the dibasic amino acid motif present in the cytoplasmic tail. Moreover, *in vitro*binding of the cytoplasmic tails of mammalian glycosylation enzymes to another COPII protein has been reported (44). Furthermore, for mammalian cells, as well as for plants, it was found that increased glycosyltransferase or glycosidase expression resulted in Sar1p accumulation at peri-Golgi areas, presumably representing ERES (21,26). We also found accumulation of Sar1p at putative ERES upon coexpression with GnTI, which indicates the involvement of COPII proteins in ER exit of plant glycosyltransferases. The expression of the tail-mutated GnTI form did not result in any association of Sar1p with ERES or peri-Golgi areas, supporting the hypothesis that the basic amino acids present in the cytoplasmic tail of GnTI are involved in recruitment of Sar1p or other COPII components to ERES. Future experiments in our laboratory will focus on the question whether there is a direct interaction between GnTI and COPII components like it was shown for other plant proteins (45,46) and whether this interaction involves basic amino acids present in the cytoplasmic tail, as was proposed for mammalian glycosyltransferases.

## Materials and Methods

### Constructs

The GnTI CTS region was amplified with primers NtGnTI-1/-2 (for primer sequences see Table S1) from *N. tabacum*GnTI complementary DNA (cDNA) (47) using GoTaq DNA Polymerase (Promega) and inserted into *Hin*dIII/*Sal*I-linearized cloning vector puc19 to produce vector puc19Nt1. The mRFP coding sequence was amplified with Turbo Pfu polymerase (Stratagene) from pRSETB-mRFP (kindly provided by Roger Tsien, UCSD, CA, USA) using primers mRFP1/2 and cloned into the *Sal*I/*Bam*HI site of puc19Nt1 (puc19Nt1-mRFP). The GnTI-CTS-mRFP coding sequence was excised with *Xba*I/*Bam*HI and ligated into the binary plant expression vector pPT2 (31). In pPT2, the expression of the DNA sequences is under the control of the cauliflower mosaic virus 35*S*promoter. The CT-mRFP and T-mRFP constructs were generated by ligation of overlapping primers NtGnTI-11F/-11R and NtGnTI-12F/-12R, respectively, into *Xba*I/*Kpn*I-digested vector pPFH3, which is a derivative of pPT2 (16). To construct the tail-mutated GnTI forms, the cytoplasmic region was excised from GnTI-CTS-mRFP with *Xba*I/*Kpn*I, and overlapping primers NtC3F/R (GnTI-C_RAA_TS-mRFP), NtC4F/R (GnTI-C_AAR_TS-mRFP) and NtC8F/R (GnTI-C_AAA_TS-mRFP) were inserted.

To generate the GMII constructs, the C_10_TS region was amplified by polymerase chain reaction (PCR) from the *A. thaliana*GMII cDNA (14) using oligos Ath-MII-40F/-44R, the C_10AAA_TS-region was amplified with Ath-MII-41F/-44R and the C_10AKA_TS-region was amplified with Ath-MII-48F/-44R. The C_10_T region was amplified using primers Ath-MII-40F/-42R and C_10AAA_T with Ath-MII-41F/-42R. All PCR products were *Xba*I/*Bam*HI digested and inserted into p20F (48) to generate the GFP fusion constructs (p20F-GMII-C_10_TS, -GMII-C_10AAA_TS, -GMII-C_10AKA_TS and -GMII-C_10AAA_T) or into p23 (48) to generate the binary vector for expression of the CFP fusion (p23-GMII-C_10_T).

The glycoprotein–GFP vectors were generated by PCR amplification of a human IgG1 heavy chain fragment from vector pTRAp-2G12-Ds (49) using primers Fc-1F/-1R. The PCR product was *Bam*HI/*Bgl*II digested and ligated into *Bam*HI-linearized vector p20F to create vector p20F-Fc. GnTI-CTS was amplified using primers NtGnTI-13F/-16R and cloned into the *Xba*I/*Bam*HI site of p20F-Fc. The cytoplasmic-domain-swap vectors were generated by *Xba*I/*Kpn*I excision of the GnTI-C-tail and insertion of NtC8F/R (GnTI-C_AAA_TS) and XT-C1F/R overlapping primers (XylT-C-GnTI-TS) or the PCR product from *A. thaliana*genomic DNA using primers GCSI-1/-2 (GCSI-C-GnTI-TS). GCSI-CTS-GFPglyc was generated by PCR amplification from *A. thaliana*genomic DNA using oligos GCSI-1/-3 and cloned into the *Xba*I/*Bam*HI site of p20F-Fc. GnTI-C-GCSI-TS-GFPglyc was generated by replacing the GnTI-TS region with a PCR fragment containing the coding sequence for the GCSI TS region obtained by using primers GCSI-4/-3.

The full-length GnTI-GFP expression vector was generated by PCR using primers NtGnTI-13F/-15R and vector pNT-GnTI-Mut as template and inserted into the *Xba*I/*Bam*HI site of p20F. Vector pNT-GnTI-Mut was generated by site-directed mutagenesis of the *Kpn*I site at position 1272 of the tobacco GnTI coding sequence in vector p5/2 (47) using a quick change kit (Stratagene) and primers NtGnTI-14F/-R. Subsequently, the cytoplasmic tail was removed and the tail-mutated form inserted as described for the mRFP and glycoprotein–GFP fusion constructs to generate GnTI_AAA_-GFP.

To generate the mRFP-HDEL vector (pmRFP-HDEL), mRFP was amplified using primers mRFP5-F/6-R and ligated into *Bam*HI/*Sal*I-digested vector p20ChisSP (unpublished data), which is derived from pPT2 and contains a chitinase signal peptide.

### Plant material and transient expression system

Four-week-old to 5-week-old *N. benthamiana*plants were used for *Agrobacterium tumefaciens*(strain UIA143)-mediated transient expression of indicated constructs using the agroinfiltration technique described previously (50,51). The optical density (OD_600_) of the bacterial suspensions used for plant transformations was 0.03 for all constructs except for ST–GFP/mRFP 0.05, ERD2-GFP 0.08, full-length GnTI-GFP and GnTI_AAA_-GFP 0.05. Constructs expressed in Sar1p experiments were infiltrated at the following OD_600_: ST–GFP 0.2, ERD2-GFP 0.2, Sar1-YFP 0.05, untagged Sar1[H74L]p 0.03 and GnTI-CTS-GFP 0.2.

### Sampling and imaging

Sections of transformed leaves were analysed 2–4 days postinfiltration (dpi) on a Leica TCS SP2 or Zeiss LSM 510 confocal microscope both equipped with ×63 and ×100 oil immersion objectives using appropriate filters or spectral selections. Images presented in this manuscript were taken at 2 dpi, unless stated otherwise. For ease of imaging motile structures, leaf segments were incubated with n-ethylmaleimide (Sigma; stock solution, 1 min dimethyl sulphoxide) used at a concentration of 50 mmfor 10 min before confocal analysis (27). The imaging settings were identical throughout experiments so that the images were comparable. To exclude the possibility of cross-talk between fluorophores, appropriate controls were performed. Post-acquisition image processing was performed in ImageJ and Adobe Photoshop CS.

Imaging with Zeiss microscope: GFP alone or in combination with YFP was imaged as described recently (27). For imaging mRFP constructs, mRFP was excited with the 543-nm helium/neon laser line, and fluorescence was detected using a 488/543-nm dichroic beam splitter (DBS) and 585/615-nm band pass filter (BPF) in the single-track facility of the microscope. YFP was monitored using a 488-nm argon laser line; fluorescence was detected using a 488/543-nm DBS and a 505/550-nm BPF. For imaging coexpression of mRFP and YFP constructs, the 488-nm argon laser line for YFP and the 543-nm helium/neon laser line for mRFP were used alternately with line switching using the multi-track facility of the microscope. Fluorescence was detected using a 488/543-nm DBS and a 505/550-nm BPF for YFP and 585/615-nm BPF for mRFP. For imaging expression of GFP constructs, the 488-nm argon laser line was used to excite GFP, and fluorescence was monitored with a 488/543-nm DBS and 505/530-nm BPF in the single-track facility of the microscope. For imaging expression of GFP in combination with mRFP, the 488-nm argon laser line for GFP and the 543-nm helium/neon laser line for mRFP were used alternately with line switching using the multi-track facility of the microscope. Fluorescence was detected using a 488/543-nm DBS and a 505/530-nm BPF for GFP and 585/615-nm BPF for mRFP.

Imaging with Leica microscope: GFP alone was imaged using a 488-nm argon laser line, and emission was recorded from 500 to 535 nm. mRFP alone was excited with a 543-nm helium/neon laser line, and emission was collected at 600–630 nm. CFP alone was imaged using a 458-nm argon laser line, and emission was detected at 465–495 nm. YFP was excited with a 514-nm helium/neon laser line and detected at 520–600 nm. For imaging GFP in combination with YFP, GFP was imaged using a 458-nm argon laser line and its emission was recorded from 475 to 510 nm, while YFP was excited using a 514-nm argon laser line and its emission was collected at 560–615 nm. Images were acquired separately and superimposed in ImageJ. Dual-color imaging of cells expressing both GFP and mRFP was performed simultaneously using a 488-nm argon laser line and the 543-nm helium/neon laser line. GFP emission was recorded at 500–535 nm, whereas mRFP fluorescence was detected at 600–630 nm. For imaging cells coexpressing GFP and CFP, scans optimized for each fluorophore were collected separately and superimposed afterwards. GFP was excited with a 514-nm argon laser line and emission detected at 520–540 nm. CFP was excited with a 458-nm argon laser line, and signal was collected at 465–495 nm. When CFP and mRFP were expressed together, imaging was performed using a 458-nm argon laser line and a 543-nm helium/neon laser line. Fluorescence signals were monitored separately, and CFP emission was collected at 465–495 nm, whereas mRFP was recorded at 600–630 nm.

For quantifying the number of Golgi stacks, 4-μm thick three-dimensional z-stack images (no cropping) were captured with a ×40 oil immersion objective of a Zeiss LSM 510 using identical settings throughout the experiment. Per experiment, eight z-stacks were analysed (three experiments in total). In ImageJ, a maximum intensity projection of each stack was made. To separate touching Golgi stacks as different objects, projections were thresholded manually, converted into binary images and further segmented using the ‘Watershed’ segmentation tool. Finally, the number of Golgi stacks was determined using ‘Analyse Particles’ with the following parameters input: size ‘0–3.643’ (area of largest Golgi stack measured in square pixels), circularity ‘0–1.0’ default, and show ‘outline’.

### Protease protection assays

Microsomal fractions were prepared from 500 mg infiltrated *N. benthamiana*leaves as described previously (52). Pellets were resuspended in 100 μL 25 mmTris/HCl (pH 7.2) and 0.5 mmdithioerythritol (DTE), and 8.5 μL was incubated in the presence or absence of 1% Triton-X-100 with 0.5 μg trypsin (Sigma) for 60 min at 37°C. To stop the reaction, 20 μg soybean trypsin inhibitor (Sigma) was added and samples were subjected to SDS–PAGE and immunoblotting using rabbit anti-RFP (US Biological) or mouse anti-GFP (Roche) antibodies for detection.

### Purification of CTS-GFP fusion proteins

Purification of the CTS-GFP fusion proteins (as listed in Figure 1C) was performed using a modified version of a previously described protocol for antibody purification from infiltrated *N. benthamiana*leaves (53). Briefly, leaves of 6-week-old plants were infiltrated with agrobacteria diluted with infiltration buffer to an OD_600_ of 0.2. For purification, 800 mg infiltrated leaf material was homogenized in liquid nitrogen, resuspended in 8 mL of precooled extraction buffer [1× PBS buffer, pH 7.2, 1% Triton-X-100 and protease inhibitor cocktail (Sigma)] and incubated for 15 min at 4°C. Insoluble material was removed by several centrifugation steps at 5000 × ***g***for 10 min at 4°C. The clear supernatant was incubated with 40 μL rProtein A–Sepharose (GE Healthcare), and the fusion proteins were eluted by boiling in Laemmli sample buffer. Protein gel blot analysis of crude extracts or purified proteins was performed using anti-HRP antibodies as described previously (54), mouse anti-GFP antibodies (Roche) or peroxidase-conjugated concanavalin A (Sigma).

### LC-ESI-MS analysis of tryptic glycopeptides

To analyse the *N*-glycans present on the CTS-GFPglyc glycoreporters, purified protein (500 ng) was separated by SDS–PAGE (10%) under reducing conditions, and polypeptides were detected by Coomassie Brilliant Blue staining. The corresponding band was excised from the gel, destained, carbamidomethylated, in-gel trypsin digested and analysed by LC-ESI-MS as described recently (53).

## Supporting Information

Figure S1: Time–course of *N. benthamiana *leaf epidermal cells expressing wild-type and tail-mutated GnTI chimeras.

Figure S2: Golgi concentration of XylT depends on basic residues within the cytoplasmic tail.

Figure S3: The subcellular localization of wild-type and tail-mutated full-length GnTI fusion proteins is identical to their GnTI-CTS forms.

Figure S4: MALDI-TOF-MS analysis of transformed *cgl1 *plants.

Table S1: Primer sequences cited in *Materials and Methods*
