# Supplementary material for: Arginine/Lysine Residues in the Cytoplasmic Tail Promote ER Export of Plant Glycosylation Enzymes
Source: Traffic. 2008 Nov 3;10(1):101–15. doi: 10.1111/j.1600-0854.2008.00841.x (PMC3014094; doi:10.1111/j.1600-0854.2008.00841.x)
Supplement: Supplementary file 1 [file tra_841_SD1.pdf]

Name	Sequence
Ath-MII-40F	ATCTAGAATGCCACGAAAACGAACTCTCGTAG
Ath-MII-41F	ATCTAGAATGCCAGCAGCAGCAACTCTCGTAGTC
Ath-MII-42R	AGGATCCGAGAGTGAGGAGGAAGAAGA
Ath-MII-44R	AGGATCCCCGATTGGATCTGGAGGTAAG
Ath-MII-48F	ATCTAGAATGCCAGCAAAAGCAACTCTCGTAGTC
Fc-1F	TATAGGATCCGCACCTGAACTCCTGGGGGGAC
Fc-1R	TATAAGATCTTTTACCCGGAGACAGGGAGAGGCTC
GCSI-1	ATCTAGAATGACCGGAGCTAGCCGTCGGAGC
GCSI-2	TGGTACCGGAGATTCGTCTTGAAAGCACCG
GCSI-3	TGGATCCAAAAGGAGTGATAACCCTAGGCTTC
GCSI-4	TGGTACCTATTGGTTGGGTTAATCATATTAGGG
mRFP1	TATAGTCGACATGGCCTCCTCCGAGGACGTC
mRFP2	TATAGGATCCTTAGGCGCCGGTGGAGTGG
mRFP3	TATAGGTACCATGGCCTCCTCCGAGGACGTC
mRFP5-F	TATAGGATCCGCCTCCTCCGAGGACGTCATCAAG
mRFP6-R	TATAGTCGACTTATAATTCATCATGGGCGCCGGTGGAGTGGCGGCCCTCG
NtC3F	CTAGATGAGAGGGTACGCGTTTTGCTGTGATTTCGCGTAC
NtC3R	GCGAAATCACAGCAAAACGCGTACCCTCTCAT
NtC4F	CTAGATGGCAGGGTACGCGTTTTGCTGTGATTTCCGGTAC
NtC4R	CGGAAATCACAGCAAAACGCGTACCCTGCCAT
NtC8F	CTAGATGGCAGGGTACGCGTTTTGCTGTGATTTCGCGTAC
NtC8R	GCGAAATCACAGCAAAACGCGTACCCTGCCAT
NtGnTI-1	TATAAAGCTTCTAGATGAGAGGGTACAAGTTTTG
NtGnTI-11F	CTAGAATGAGAGGGTACAAGTTTTGCTGTGATTTCCGGTACCTCCTCATCTTGGCTGCTGTCGCCTTCATCTACATACAGATGCGGCTTTTTAGTAC
NtGnTI-11R	TAAAAAGCCGCATCTGTATGTAGATGAAGGCGACAGCAGCCAAGATGAGGAGGTACCGGAAATCACAGCAAAACTTGTACCCTCTCATT
NtGnTI-12F	CTAGAATGTACCTCCTCATCTTGGCTGCTGTCGCCTTCATCTACATACAGATGCGGCTTTTTGGTAC
NtGnTI-12R	CAAAAAGCCGCATCTGTATGTAGATGAAGGCGACAGCAGCCAAGATGAGGAGGTACATT
NtGnTI-13F	TATATCTAGAATGAGAGGGTACAAGTTTTG
NtGnTI-14F	GTGGTTTTCCGGTATCAAACGTCCAGAC
NtGnTI-14R	GTCTGGACGTTTGATACCGGAAAACCAC
NtGnTI-15R	TATAGGATCCAGTATCTTCATTTCCGAGTTGTT
NtGnTI-16R	TATAGGATCCCTGACGCTTCATTTGTTC
NtGnTI-2	TATAGTCGACCTGACGCTTCATTTGTTC
XT-C1F	CTAGAATGAGTAAACGGAATCCGAAGATTCTGAAGTAC
XT-C1R	TTCAGAATCTTCGGATTCCGTTTACTCATT

Supplementary Table S1

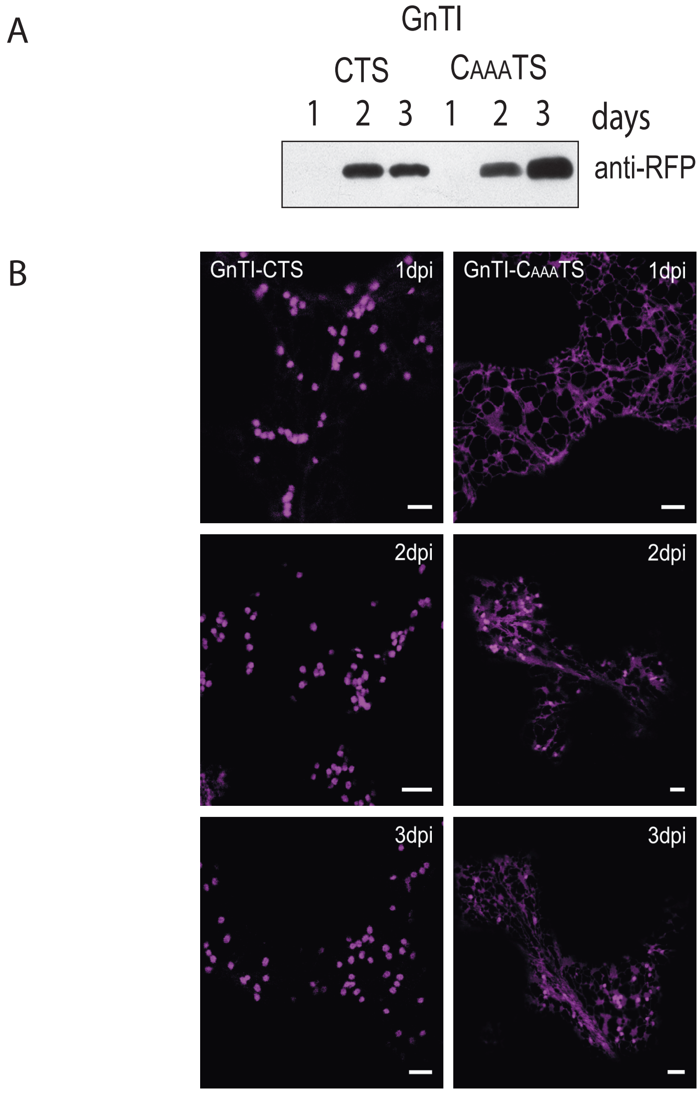

Supplementary Figure S1. Time-course of *N. benthamiana* leaf epidermal cells expressing wildtype and tail-mutated GnTI chimeras. (A) Western blot analysis of *N. benthamiana* leaf epidermal cells expressing GnTI-CTS-mRFP and the tail mutated form GnTI-C_{AAA}TS-mRFP. Proteins were extracted 1 to 3 days postinfiltration, subjected to SDS-PAGE (12%) and analyzed by immunoblotting using anti-RFP antibodies (B) GnTI-CTS-mRFP and GnTI-C_{AAA}TS-mRFP were expressed in *N. benthamiana* leaf epidermal cells and imaged 1 dpi, 2 dpi and 3 dpi. The wildtype GnTI fusion predominantly localized to Golgi stacks throughout the entire time course. The tail-mutated GnTI fusion predominantly marked the ER with a slight increase of Golgi labelling over time. Scale bar = 5 µm for all images.

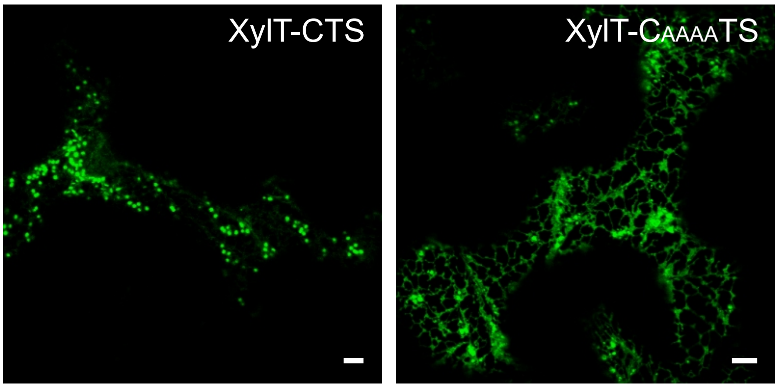

Supplementary Figure S2. Confocal images of wildtype and tail-mutated XylT-GFP transiently expressed in *N. benthamiana* leaf epidermal cells. The wildtype XylT-CTS-GFP fusion targets its steady-state location, which is the Golgi apparatus. Note the faint ER staining that occasionally showed up. Similarly to the tail-mutated forms of GnTI and GMII, XylT-C_{AAAA}TS-GFP fails to exit from the ER, which points out the importance of basic amino acids in the cytoplasmic tail to ER export and Golgi localization. Scale bar = 5 µm for all images.

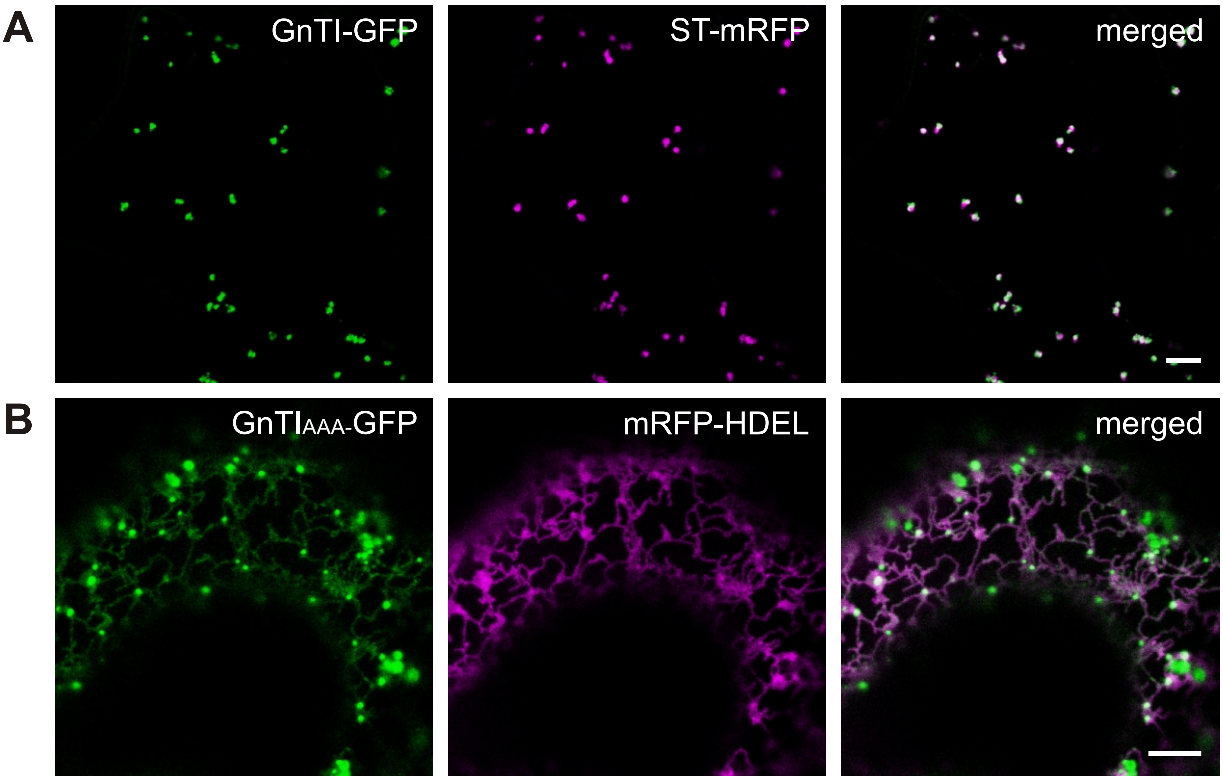

Supplementary Figure S3. The subcellular localization of wildtype and tail-mutated full-length GnTI fusion proteins is identical to their GnTI-CTS forms. A) Confocal images of a *N. benthamiana* leaf epidermal cell coexpressing full-length GnTI-GFP with the Golgi marker ST-mRFP. B) Images of full-length $GnTI_{AAA}$-GFP coexpressed with the ER marker mRFP-HDEL. Scale bar = 5 µm for all images.

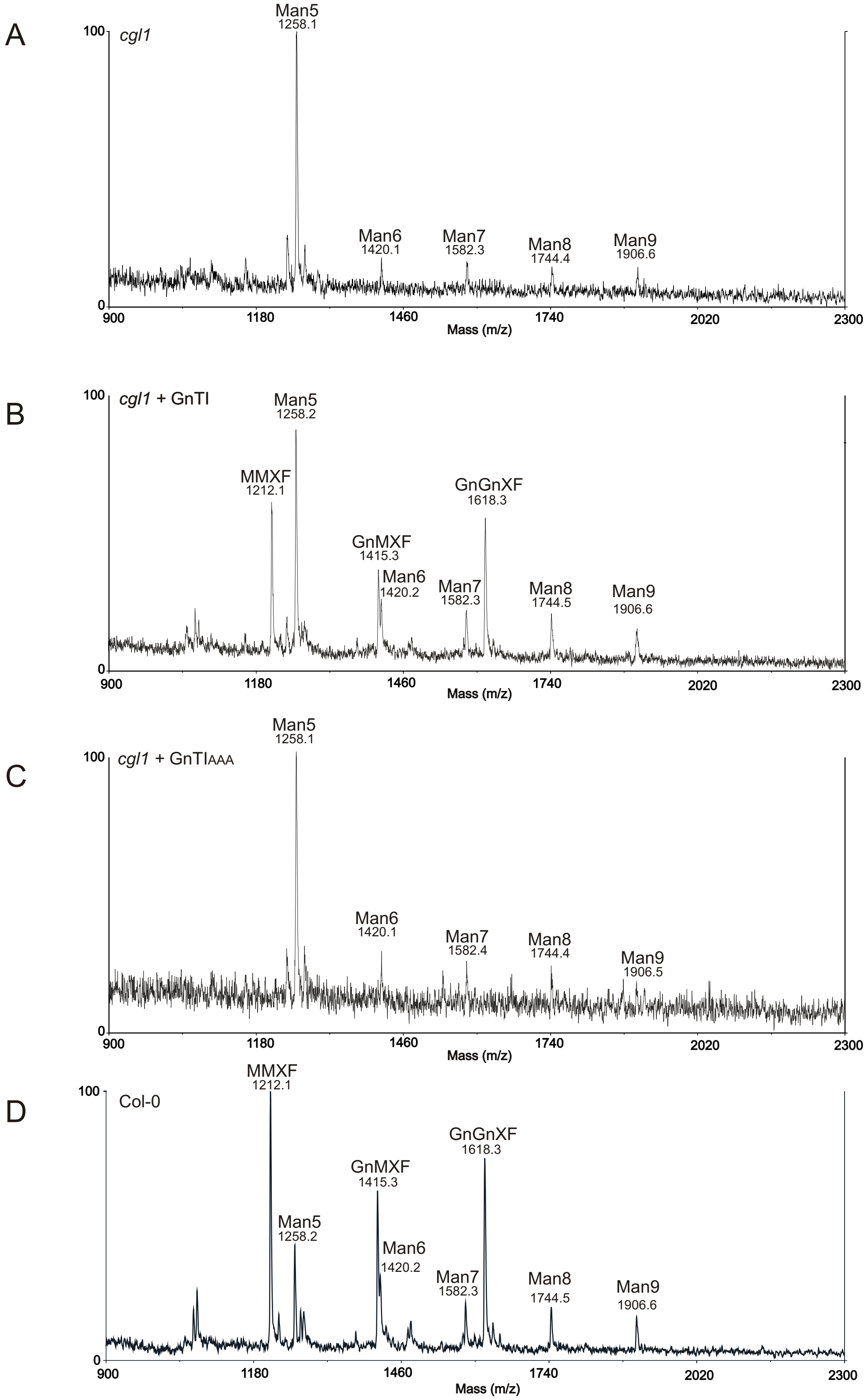

Supplementary Figure S4: Matrix-assisted laser desorption ionization-time of flight/time of flight mass spectrometry (MALDI-TOF-MS) analyses of total N-glycans extracted from leaves of transformed *cgl1* plants. Analysis was done as described previously (Strasser et al., 2005). (A) *cgl1*: untransformed control displays oligo-mannosidic N-glycan structures as described previously (von Schaewen et al., 1993; Strasser et al., 2005). (B) *cgl1* + GnTI-GFP (line 6) shows the formation of complex N-glycan structures (MMXF, GnMXF, GnGnXF), but the N-glycan pattern is not completely restored compared to wildtype plants. (C) *cgl1* + $GnTI_{AAA}$-GFP (line 8) shows no detectable complex N-glycans, indicating the absence of GnTI in the Golgi apparatus. (D): Col-0: N-glycan structures found in wildtype plants. See http://www.proglycan.com for an explanation of N-glycan abbreviations.
